# Supplementary material for: Effects of Perennial Alfalfa on the Structure and Function of Soil Micro-Food Webs in the Loess Plateau
Source: Microorganisms. 2024 Nov 8;12(11):2268. doi: 10.3390/microorganisms12112268 (PMC11596237; doi:10.3390/microorganisms12112268)
Supplement: Supplementary file 1 [file microorganisms-12-02268-s001.zip › microorganisms-3292853-supplementary.pdf]

S1. Soil physicochemical properties of alfalfa fields with different planting years

Item	CK	2years	9years	18years
Bulk density ($g\cdot cm^{-3}$)	1.18±0.01a	1.21±0.01a	1.23±0.02a	1.25±0.03a
Soil water (%)	20.49±0.12a	15.91±1.02b	14.50±0.28b	14.24±0.21b
pH	8.42±0.06a	8.58±0.07a	8.65±0.06a	8.55±0.07a
Total nitrogen ($g\cdot kg^{-1}$)	0.98±0.04c	0.96±0.05c	1.13±0.01b	1.28±0.02a
Total phosphorus ($g\cdot kg^{-1}$)	0.82±0.01a	0.74±0.01bc	0.76±0.01b	0.72±0.01c
Available phosphorus ($mg\cdot kg^{-1}$)	13.39±0.43a	7.98±0.18b	7.09±0.05c	6.99±0.09c
Organic carbon ($g\cdot kg^{-1}$)	9.86±0.11b	9.79±0.14b	10.48±0.26b	11.65±0.30a

Note: values are mean ± SE in triplicate replicates.
